# Supplementary material for: Impaired Performance of Broiler Chickens Fed Diets Naturally Contaminated with Moderate Levels of Deoxynivalenol
Source: Toxins (Basel). 2021 Feb 22;13(2):170. doi: 10.3390/toxins13020170 (PMC7926331; doi:10.3390/toxins13020170)
Supplement: Supplementary file 1 [file toxins-13-00170-s001.pdf]

Supplementary Materials: Impaired Performance of Broiler Chickens Fed Diets Naturally Contaminated with Moderate Levels of Deoxynivalenol

Regiane R. Santos and Ellen van Eerden

Table S1. Composition of the experimental diets.

Ingredients (%)	Starter D0-14	Grower (D14-28)	Finisher (D28-35)
Wheat (with moderate or low DON)	55.00	55.00	-
Wheat (with marginal DON)	-	-	44.96
Soybean meal	24.97	19.98	18.24
Rye	5.00	7.50	-
Oats	-	-	3.98
Peas	-	-	3.25
Rapeseed meal	1.00	2.50	-
Corn	1.06	3.31	11.00
Corn gluten meal	-	-	6.00
Sunflower seed meal	3.76	2.64	3.98
Soya oil	1.02	0	0.75
Poultry fat	3.98	5.55	3.00
Palmkernel fatty acids	-	-	1.50
Salt	0.11	0.12	0.08
Limestone	1.16	1.04	0.72
Monocalcium Phosphate	1.22	0.74	0.19
Sodium Bicarbonate	0.35	0.30	0.33
Lysine HCl	0.39	0.37	0.31
DL-Methionine	0.30	0.25	0.21
Threonine	0.13	0.12	0.11
Valine	0.04	0.04	0.35
Arginine	0.02	0.06	-
Vitamin & Mineral premix	0.50	0.50	0.40
Phytase 1000 FTU	-	-	0.30
Ammomin	-	-	0.48
Choline-chloride	-	-	0.02
**Total**	**100.00**	**100.00**	**100.00**
**Nutrients**			
Energy. kcal/kg	2,875	2,950	3,090
DM. g/kg	883	882	887
Ash. g/kg	55	47	44
Crude protein. g/kg	215	196	183
Crude fat (acid hydrolysis). g/kg	72	77	71
Crude fibre. g/kg	28	28	34
Ca. g/kg	7.6	6.3	4.9
P. g/kg	6.6	5.3	4.2
K. g/kg	8.9	8.0	7.7
Na. g/kg	1.5	1.4	1.3
Cl. g/kg	2.0	2.0	1.8
